# In Between New Public Management and Network Governance in Austria, Finland and Scotland: Potential Conflicts in Autonomy Understandings of Governments and Universities

**DOI:** 10.1007/s13132-021-00881-z

**Published:** 2022-03-09

**Authors:** Kajetan Stransky-Can

**Affiliations:** University of Vienna, Vienna, Austria

**Keywords:** Comparative political science, Epistemic governance, Higher education governance, Network governance, New public management, University steering

## Abstract

The political science doctoral thesis project is focussing on comparing university policies with the background of different welfare state types: Austria as a conservative-corporatist, Finland as a social democratic and Scotland as a liberal welfare state. As universities are pre-constitutional entities, it seems to be legitimate to search for fit from welfare state policy-making with the practices of university steering. Here, I will present paradigm evidence of top-down-oriented New Public Management (NPM) and bottom-up-formed Network Governance (NG) at a macro-level, i.e. governance by the state. The paradigms connect with the theme of institutional autonomy, which differentiates into an academic, a financial, an organisational and a staffing aspect. As a result, I propose the following hypothesis for the cases of Austria, Finland and Scotland: *NPM is present, but not dominant in higher education (HE) policies.* More likely, NPM and NG appear simultaneously within the university policies of Austria, Finland and Scotland. *How do these paradigms transfer within the institutions?* In this article, a concept for this transfer of action in horizontal autonomy to actors in vertical autonomy through communication and through management committees’ interplay elaborates and, additionally, hypotheses formulate. *The extent of the space for alternative paradigms to join the paradigm transfer game, for example, for Epistemic Governance (EG), might be larger, the higher the regulative density of university policies is.* The borders of every paradigm in university policy, however, constitute with (a) strong welfare state traditions for governance and (b) strong university traditions for steering. Conservative-corporatist welfare state tradition as in Austria shows an inclination for university policy governance to be control-oriented, whereas social democratic welfare state tradition as in Finland relies on trust in university policy formulation (and implementation). It has to remain open how the liberal welfare state of Scotland would classify.

## Outline, Background^1^

The cumulative doctoral thesis with its working title “An Analysis of the ‘Knowledge State’ in Austria (AT), Finland (FI) and Scotland (SCO): The Example of Higher Education Policy – Expertise from Universities and their Institutional Environment” is using framing (Entman, 1993) analysis of university policies for drawing conclusions on paradigms and themes used at the macro-level, which is university governance. Data from problem-centred interviews (Witzel & Reiter, 2012) enrich these results. This contribution builds on a conceptual approach to differentiate governance and steering^2^ levels (Stransky-Can, 2019b, 189–190) for identifying positions in autonomy understandings of governments and universities. As regarding the thematic understanding of the paradigms within university policies in the three countries, the analysis for AT from Stransky-Can and Campbell (2021) extends to include FI and SCO.

The question, whether there are differences between the internal perceptions at higher education (HE) institutions and the external perceptions of governance of the HE systems, is of special interest here. First, a theoretical framework in differentiating between horizontal autonomy (i.e.: What is the range of manoeuvre of universities within their legal framework?) and vertical autonomy (i.e.: Which room of manoeuvre is remaining for management to steer the political system of a university?) is presented. Second, a concept of operationalizing the differences in autonomy understandings in horizontal and vertical autonomy outlines for AT, FI and SCO. For being analytically clear, the distinction between macro-, meso- and micro-levels (Zechlin, 2018) has been useful, whereas the first—for governance—are the focus of this paper. The results of presenting the theoretic framework show in (sequentially decreasing) distance to the meso- and micro-levels of university steering, whereas the concept of operationalizing differing autonomy understandings shall be more flexible in this regard. Finally, there is direction to answering the following question: Are the two paradigms of top-down New Public Management (NPM) and bottom-up Network Governance (NG) still sufficient for understanding University Policy or is there an alternative paradigm developing?

The selection of country cases comes from the background of ideal types of welfare state regimes (Esping-Andersen, 1990), “Austria (as an Esping-Andersen type of conservative-corporatist with a relatively high government expenditure ratio), Finland (as social-democratic with a highest government expenditure ratio) and Scotland (as liberal with a low government expenditure ratio). Those three HE systems are, nevertheless, of comparable size and interconnected by the European politics level (e.g. EEA, ERA)” (Stransky-Can & Campbell, submitted, 2). The last point seems to be of increasing importance for the fiscal role of the European Union (EU) in the current situation coping with the COVID-19-pandemic (Stransky-Can, forthcoming). AT is seen as a case of a conservative welfare state where HE policy has undergone significant change to another governance regime in 2002 (resolution of the University Act–Universitätsgesetz, 2002 [UG]), with preparations in partly autonomous institutions reaching back to at least 1993 (Universitätsorganisationsgesetz 1993 [UOG, 1993]).^3^ This country should be in an economic “high-tech-state” now where technological progress and higher qualifications are the main drivers of economic growth (Aiginger et al., 2006, 5). FI, as an example of a social democratic welfare state, has followed some elements of the UG to implement changes in governance of HE policy roughly ten years ago (Winckler, 2014, 110).^4^ A “new law was drawn up over 2008–2009” with an “aim […] to increase autonomy for universities” (Broucker et al., 2015, 7), as autonomy has principally already been codified in 1997.^5^ Similar to the governance framework (not to the functioning of steering) in AT with the “Rektorat”, the “Senat” and the “Universitätsrat”, the bodies of a rector and a board manage universities in FI (ibid., 18). SCO as a case for a liberal welfare state has passed the Higher Education Governance (Scotland) Act in 2016. Here, the governing body and the principal constitute the institutional executive management. Furthermore, it seems to be one of the first countries in Europe where “the governments aimed at making universities more accountable for the funding they received, but also restraining detailed regulation in favor of market-like mechanisms.” (De Corte, 2014, 133).^6^

## Research Questions^7^

The questions presented in this section are those, which make up the cumulative dissertation project. Note, however, that the focus within this paper lays on question b), exploring the relationship between macro- and meso-levels at universities from a macro-perspective, whereas the main question of the whole dissertation project is question a), looking at the thematic understandings of the paradigms of NPM and NG, which will address to a lower extent within this paper. The answer to this question for AT in concept and empirics gives Stransky-Can and Campbell (2021; submitted; with a focus on empirics, see also Stransky-Can, 2019b, c) and extends, here, to FI and SCO. Therefore, the comparison of the countries presents here as new, but it conducts within an established framework. The treatment of question c) will proceed here to a similar extent as in the whole dissertation project, with the aim of outlining starting points for further research.

### a) What Is the Autonomy Aspect Understanding of the Paradigms of NPM and NG Within Higher Education (HE) Policies of AT, FI and SCO and How Do These Paradigms Relate to Each Other?

As a simplified welfare state categorization might suggest, the themes as defined with the European University Association (EUA-) autonomy scorecard dimensions show indeed that SCO as a part of the UK (minimum state) has the most autonomous universities. FI (maximum state) and AT (intermediate state) follow, whereas, notably, FI is coming close or even surpassing the UK (excluding the financial autonomy scores, where the UK is ranked clearly ahead). AT universities do not have similar extents of autonomy in all four dimensions (EUA, 2021a). In the classification by Aghion et al. (2007, 5), UK institutions have, compared to AT’s neighbouring country Germany and FI’s neighbouring country Sweden, more budget autonomy and building ownership possibilities. For the UK, this is consistent with the EUA scores on financial autonomy (which includes building ownership). In hiring autonomy, however, Swedish institutions reach the same score as UK institutions (with Germany’s coming close), and on wage-setting autonomy, the former are even ahead of the latter. Excluding Germany, these results are comparable to the EUA scores, too, in this case for staffing autonomy. The different positions of AT and FI within this dimension are either reflecting the more intensive implementation efforts of the above-mentioned reforms in FI compared to AT, although there is indication that there has been a connection between the latest fundamental HE policy reforms in both countries. Or the differing starting points in codified full institutional autonomy (1997 and 2002, resp.) may reflect in these different scoring positions. All over, the publication by Aghion et al. (2007) could have pointed to a more narrow understanding of university autonomy, since it includes only financial and staffing aspects. Nevertheless, these early identified aspects underscore the EUA indicators^8^ as they show similarities in empirical results for countries clustering within different welfare state regimes. Regarding the EUA autonomy scores, we can summarize that a gap of AT to the Anglo-Saxon resp. Scandinavian country is somehow identifiable, whereas FI seems to converge to the UK (and even surpassing it in academic autonomy).

### b) Are There Differences Between the Internal Perceptions (at Universities) and the External Perceptions of Governance of the HE Systems?

SCO as an initially liberal welfare state could be a first mover among the nations under investigation in adopting hard accountability to its HE policies as a feature of NPM (see the “Outline, Background” section). As Stransky-Can (2019a, 2) puts it: “Practically, the Anglo-Saxon countries, including the UK, have been implementing NPM in public administration from 1980 onwards, followed by countries in Northern Europe including FI and German-speaking countries including AT from 1990 onwards”. The implementation of NPM in these parts of the continent proceeds by “issue-oriented coordination”, “dialogue and change process”^9^ and “outcome-oriented steering”, respectively (ibid., 3). For university steering, are these approaches applicable (or applied)? As a first step, the two paragraphs within this sub-section illustrate these narratives for AT, FI and SCO. Outcome-oriented steering, indeed, has been a major driver for budget reform in AT (Steger, 2014, 92–96). There, it also might have been the narrative for NPM-guided university policy reform of 1993 (Stransky-Can, 2019c).

Regarding a status quo and already at a first glance, one could find elements of both NPM and NG also in Finnish and Scottish law such as the Universities Act of 2009^10^ and the Higher Education Governance (Scotland) Act of 2016,^11^ respectively. As the consultation process with the universities on the Scottish bill since 2011 indicates, the formulations in law could be compromising (which is a hint to bottom-up NG) or non-compromising (which is a hint to top-down NPM) with internal (HEIs’) interests. The formulations might also connect to a discursive understanding of institutional autonomy: “[T]he more consensus and common understanding, the more willingness there may be to leave decisions to universities and to higher education as a policy sector. The more conflict there is, the more likely [sic!] that there will be competing demands for representation and participation in reform processes and in University governance in general.”(Olsen & Maassen, 2007, 22) Overall, the EU is a policy-making instance with growing relevance for policy-making in nation states in many fields since at least 1992 (Kuhlmann, 2001, 957). For the EU, it makes sense to differentiate, however, between the adoption of fiscal and strategic instruments for the universities sector (even though the former ones are currently expanding – Stransky-Can, forthcoming, 2). The long history within European frameworks for SCO, compared to AT and FI, illustrates as follows: “Scottish Qualifications Framework is strong. And a lots of European influence and the European Credit Transfer thing” (representative of the university sector SCO). As another example for the strategic importance of the EU shows, the Austrian Institute for Economic Research has recommended the establishment of a European research institute of excellence (Aiginger et al., 2006, 4). Meanwhile, this has realized with founding the European Research Council in 2007 within the prevalently supranational policy field of research and innovation.

### c) Are the Two Paradigms of NPM and NG Still Sufficient for Understanding University Policy or Is There an Alternative Paradigm Developing?

Knowledge democracy, understood by Campbell and Carayannis (2012, 13) as a plurality in knowledge and innovation generation forms, could go beyond NPM because it is formally non-hierarchic and beyond NG because it takes a (not necessarily coordinated) network-overarching approach in agenda setting. Therefore, it could be (non-hierarchic) network-overarching arrangements that are role models for knowledge democratic societies.

In this sub-section, first, the starting point of analysis outlines as HE policy having attributes being specific for a policy field such as themes, paradigms and problem solving mechanisms, at least at the starting time of the investigation here as of roughly 1990.^12^ In this year, Esping-Andersen (1990) has published on welfare state research that serves as substantial analytic framework here. Second, countries falling under the classic ideal types of liberal, conservative and social democratic welfare state regimes develop politically and economically since then. This development reflects in political and HE institutions. It must be investigated whether we could go so far as to classify AT, FI and/or SCO as knowledge states (Campbell, 2006, 26–27) today—a step that cannot be taken without having a more intensive look at paradigms in HE politics. University acts serve as artefacts of these political processes. This could be of interest here for (b) from the following points (ibid.):

- Politics acknowledge the status of knowledge for society, democracy and the economy.
- Politics aim at supporting knowledge.
- With knowledge and/or innovation policies, politics want to support the economic development by distributing societal knowledge to economic actors.
- In a cybernetic sense, economic development is also serving the societal knowledge stocks.
- Non-economic targets are also included into knowledge and/or innovation policies.

Stransky-Can (2019a) provides ideas of connecting these points with concrete policy outputs. For now, it seems clear that, third, in all three countries under investigation, political paradigms have been underlining HE policies. This constitutes the comparative framework for the doctoral thesis. NPM and NG, fourth, are political paradigms of relevance, but, fifth, there could be also other (emerging) paradigms.

## Operationalizing NPM, NG and Their Understandings^13^

Do welfare states under increasing fiscal constraints develop preferences for certain policy fields, for example, science policy (Campbell & Carayannis, 2016)? Hints from the universities sector to elements of a knowledge state, where politics aim at supporting knowledge (Campbell, 2006, 26–27), can be found in actor-rooted thematic understandings of policy paradigms, i.e. understandings of aspects of university autonomy. In methodological terms, the approach to identify the frames/paradigms (the last as the term for operationalization) and their thematic understandings generally and for AT, FI and SCO university policy specifically classifies here as framing (Entman, 1993) analysis.^14^ Themes (as a methodological term) define as the following aspects of university autonomy (as the operationalized term—EUA, 2021a, 7):

1. Academic autonomy means the selection of students, the designing of curricula and the quality management in research and teaching.
2. Financial autonomy covers issues like funding mechanisms from public entities, the possibility to own infrastructure and keep surpluses and to impose tuition fees.
3. Organizational autonomy is about the freedom of individual institutions in structuring their faculties and institutes as well as defining the constitution of governing bodies.
4. Staffing autonomy is relevant for recruitment, promotion and dismissal procedures.^15^

The frames are the paradigms of NPM and NG in higher education (HE—Ferlie et al., 2008). NPM applied to the HE system has the “following signs and symptoms” (ibid., 335–336):

1. Stimulation of competition for research funding.
2. Policy stress on diversity and choice, including real prices of teaching fees.^16^
3. A hardening of soft budgetary constraints.
4. Explicit measurement and monitoring of performance with audit and checking features.
5. Performance contracts signed by strong rectorates.
6. A reduction in the representation of faculty in HE institutions’ governance.
7. Stronger and more overt managerial roles by senior academics.
8. Private sector style human resource management.

Regarding “policy and management implications for the HE sector, the network-governance narrative [here: paradigm] implies” (ibid., 337–338):

1. A substantial self-steering and self-organizing capacity.
2. Networks focussing explicitly on […] forms of “organizational learning”.
3. “External control systems” fall in line with “light touch systems” and “professional self-regulation”.
4. An inclusion of “softer leadership skills”, “distributed leadership” and “team-based approaches”.

**Fig. 2:**
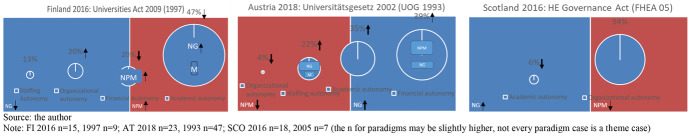
Results of comparison of university policies by framing

On point 2 here, see the cross-connection to organizing networks in knowledge-democratic environments (see above, the “Research Questions” section). Applied to AT, Stransky-Can (2019c) shows that NPM and NG were and are both relevant for university policies, although—concerning their thematic categorization—only financial autonomy can be identified as constantly relevant (between 32 [1993] and 41% [2018] of the sum of NPM and NG cases). The “Results” section adds the framing analysis results on FI and SCO (Fig. 2).

## Theorizing on Differences Between External and Internal Perceptions of Governance

### a)^17^ Understanding the Themes of University Autonomy

The map in Fig. 1 visualizes two fundamental propositions that guide the analytic view towards university governance. First, the levels of governance (steering) define the layers on the vertical axis. Governance immediately concerns the interplay of the state and the institutions, i.e. the macro-level. For steering, a certain extent of horizontal institutional autonomy is available for university management committees^18^ that take measures to translate the requirements from horizontal autonomy into institutional autonomy. We see this by moving on the horizontal axis towards the intersection of institutional and actor factors in university governance and steering. According to the horizontal-institutional translation, university management takes action to steer on the meso-level (intermediate level), i.e. executing the possibilities of vertical institutional autonomy,^19^ given the framework from horizontal autonomy. It understands this framework in terms of the meso- and micro-founded aspects of university autonomy, being the academic, financial, organizational and staffing themes (on the far right).^20^

Two-sided vertical arrows illustrate trade-off relationships—these can affect the relationship between horizontal and vertical as well as between vertical and scientific autonomy and between institutional and individual (collective) autonomy. The two-sided horizontal arrows within the aspects of university autonomy illustrate potential (reinforcing or trading-off) relationships between the aspects, with (lagged) direct consequences from horizontal for vertical autonomy, as shown in the “Results” section.^21^ One-sided arrows illustrate unidirectional relations; these show either the means of information transmission on the aspects between governance and steering operations (red thin lined arrows) or the interplay of management committees within an institution (red thick lined arrows).

For an understanding of whether there are differences between the external and internal perceptions of governance of a higher education system, within the proposed framework of cross-country-cross-section comparison in university acts, the focus lays on the dynamic aspects within the analytic map. Therefore, one can look at either the means of information transmission, the interplay of the management committees and/or the relationship between horizontal and vertical autonomy. As for vertical autonomy, we need more research, but we also refer to the “Results” section.

a) a) University acts categorize as central means of formulating governance ideas, and public funding is (more or less) dominant in AT, FI and SCO universities, which sets the focus towards the means of information transmission. The difference between the instruments of acts and funding is that acts are more general in addressing the levels of governance (steering) than funding, which only addresses—from a macro-view—the university management on top as global budgets^22^ are operating. A focus on analysing primarily acts, therefore, has the advantage of not focussing too narrowly on the governance side of university politics as to not omitting its (increasingly expanding) steering function.^23^ It is the direction from the macro- to the meso-level that, from the top, comes next from a governance perspective after having made transparent the macro-level operationalization of paradigms and themes. Here, the quantitative framework from the “Operationalizing NPM, NG and Their Understandings” section extends to include qualitative information on university acts and funding from interviewees^24^ from the three countries.

a) b) The interplay of the top management committees is outlined from qualitative information, too, as to lay out future directions for institutional research (this point specifies in the “Results” section).

To sum up, the six most recent university acts address the dimension of horizontal institutional autonomy, i.e. the room of manoeuvre for a university (as the most traditional attribute of a higher education institution) within its legal framework (among others?), with the paradigms of NPM and NG. The “Results” section presents the relationship between these paradigms and hints to a clearer understanding of NPM and NG, which form (generically) the themes of university policy application at the interface of macro- and meso-levels of university governance, i.e. in the interaction between the state and the top management committees of the universities. Here, the term of institutional autonomy operationalizes, and we can look at whether there is supportive knowledge state action (see the “Research Questions” section). This is to explicate the focus of the results from framing analysis on the structure of relationship between the state and the university management. The themes are equivalent to the aspects of university autonomy, which operationalize at the meso-level by university management, i.e. in executing vertical institutional autonomy. This means the recognition of the possibilities university management layers have in organizing the political system of an institution. As an interview partner puts it: “Hochschulverwaltung ist eigentlich dafür da, das Recht zu exekutieren und das Hochschulmanagement ist da, die Freiheiten zu nutzen” (Assistant professor for research in education and further education, 2018). He further specifies that the intersection of law (administration) and strategy (management) is a source of friction that is prevailing (in AT universities).

The “Results” section is also discussing the perspective of Epistemic Governance (EG), operationalized at the interface of meso- and micro-levels, for an approach to supplement the paradigms of NPM and NG in arranging vertical institutional autonomy.

### b)^25^ Understanding the Frames of University Autonomy

As a further paradigm added to NPM and NG (as presented in the “Operationalizing NPM, NG and Their Understandings” section, EG (Campbell & Carayannis, 2013, 2016) combines bottom-up (NG-) and top-down (NPM-) processes within institutions by explicitly acknowledging the epistemic base which drives the maintenance of knowledge stocks, the knowledge creation and the knowledge use. It focuses on a democratic approach towards knowledge creation as another aspect of knowledge democracy (Carayannis & Campbell, 2012, 13) adding to the illustrated phenomenon (see "Research Questions").

The (meta-) paradigm of Epistemic Governance (EG) says:

- Governance only works when the building blocks of knowledge stocks, use and creation are acknowledged for governance formulation and implementation.
- In contrast to NPM, quality assurance is an instrument, but not the only one towards quality enhancement (comp. the principles of audits vs. evaluations).
- Knowledge creation works either in mode 1 (basic research), mode 2 (applying basic research) or mode 3 (basic and applied research parallel).^26^

## Results

Each act is subject to one frame analysis, with the meta-data presenting above (Table 1). A case defines as a paragraph or a subparagraph. Instead of using only paragraphs, the approach of including subparagraphs is relevant because a framing/theme can appear even at subparagraph-level. The thematic comparison of the paradigms of NPM and NG as their understanding in university policies presents first. In this context, it must be clear that the identification of a case as framed by a paradigm rests on a process of interpretation. From that, we see tendencies for the acts, which we can confirm or reject. This discussion expands in the remaining two sub-sections with data from qualitative interviews, conducted between spring 2018 and fall 2020.

AT and FI show comparably increasing regulative density (from different levels, though), while there is for both countries’ policies a decreasing proportion of matches for NPM and NG.^27^ Contrastingly, regulative density in SCO university policy is lower now than it has been in the past. There is a somehow higher proportion of NPM and NG matches within the Higher Education Governance (Scotland) Act 2016 than in the Further and Higher Education (Scotland) Act 2005. It has to be remembered, though, that the amendments of the AT Universities Act 2002 in 2018 and of the FI Universities Act 2009 in 2016 are (formally, not necessarily substantially) largely offsetting legislation within the here compared period (i.e. 1993 for AT and 1997 for FI), which is not the case for the mentioned legislation in SCO. There is no obvious connection between the extent of fiscal competencies of a state and the regulative density in university policies: We would have expected a positive relationship between state expenditure and regulative density. The here conducted analysis of university policies, therefore, opens for other fields of comparison for AT, FI and SCO as contrasting welfare state types.

Another element being similar for university policies in these countries are major changes in regulation shortly after millennium (running out of the laws from 1993 in AT, from 1997 in FI and from 1992 in SCO). Additionally, the factor of intergovernmental/supranational integration is not responsible for country differences from a university policy perspective: whereas SCO has been part of the European Communities/EU for the whole period of investigation; AT has begun to reform universities before and FI after joining the EU in 1995. The Bologna process might have been a turning point in all three countries for the renewed momentum in reforms of university policies. Is politics more influential for university policies than polity?^28^ For the intergovernmental level, the considered construction of the European Higher Education Area, which is also including non-EU member states, can point towards a hesitating yes. Beyond law making, politics is also involved into softer governance formulation when it comes to funding^29^ and strategies. These national politics are, as by definition, more dynamic than polity, i.e. constitutions and the subsumed law (which might be a base point for this dynamic). This is also the case for supranational politics when we look at the European framework for research and innovation. Politics are the more traditional form of governance^30^ that seems to fit pre-constitutional institutions such as universities (Kneucker, 2020, 359) the most.

### On NPM, NG and Their Understandings in University Policies of AT, FI and SCO^31^

For AT, first results on paradigms and themes in university policy present in Stransky-Can (2019c). Stransky-Can (submitted) has been a first attempt to publish on university policies in all three countries. In Stransky-Can/Campbell (submitted), the focus has been, again, on AT, with the refinement of transparent methodology and references to current projects. This article finally publishes in a German version (Stransky-Can/Campbell 2021). Therefore, it remains, here, to extend this analysis to the country cases of FI and SCO (Fig. 2).

For all university acts, the paradigms of NPM and NG prove relevant (however, for SCO 2005, with only seven cases, which seem to be low for further analysis). A light tendency towards NPM identifies for AT, in a clearly declining trend, though. In FI and SCO, bottom-up NG is (and has been) more relevant than top-down NPM. With this background, AT is the outlier concerning the paradigms. Furthermore, we could say that for FI and, more so, AT, the paradigm that is favoured is facing a declining trend over time: For the first, NPM is gaining in importance since 1997, whereas for the second, NG is much more popular than it has been in 1993. Therefore, it could be that for these two countries, there are pendulum swings in popularity of university policy paradigms, i.e. we see the extent of NPM or NG understanding on a continuum. NG is also much more popular in SCO, without a pendulum swing, however: NG is still gaining in importance here (as in contrast to England).^32^

The law of each country sets differing thematic foci. They lead us to the answer to the question, which the understanding of the paradigms is. In AT and FI, the aspects of financial and academic autonomy are key. In SCO, the dominant theme is the aspect of organizational autonomy. Against this background, SCO is the case of the thematic outlier. This hypotheses comes even more to the fore when we consider the fact that SCO has the lowest regulative density in country comparison (see also Table 1): “[C]ompared to other countries I have ever worked with, we have very little frame in terms of legal frameworks” (representative SCO). The FI and AT universities acts mention academic and financial autonomy the most, within the country comparison. Furthermore, these acts compare with the dominant framing of themes: With a declining tendency from the FI to the AT act, NG frames the academic, respectively, the staffing autonomy, whereas financial autonomy frames in both countries within NPM. In FI, this paradigm perceives as stark: “[U]niversities, […] by large, they don’t have significant more autonomy. [… A] significant part how public steers was in public funding” (adjunct professor in higher education administration, 2019). In the SCO universities act of 2016, academic autonomy plays a diminished role in comparison to the act of 2005.

**Table 1:** Regulative density of the university acts in AT, FI and SCO

Ⓟ country: years	AT: 2018/1993	FI: 2016/1997	SCO: 2016/2005*
Cases	1112/394	311/133	123/896
Therefrom, NG + NPM	29/48 (3%/12%)	16/11 (5%/8%)	18/8 (15%/1%)

Source: the author

*Further and Higher Education (Scotland) Act 1992 not included (*n* = 3)

Meanwhile, in the AT acts, an increasing importance of academic autonomy identifies, staffing autonomy, too, is more important in 2018 than it has been in 1993 (organizational autonomy, in sharp contrast to SCO, plays a marginalized role). In FI, staffing autonomy only becomes an issue in the universities act of 2016, what comes to the expense of academic autonomy when compared to 1997: “Another thing that was significant for universities […], in 2009 or as the law came into force, the universities themselves became employers.” (ibid.)

University policy in AT proves as a framing outlier, as top-down-oriented NPM matches some more cases than bottom-up-oriented NG (in FI and SCO, it is the other way around). For university policy in SCO, organizational autonomy is the most relevant theme, whereas for both AT and FI, academic and financial autonomy are the themes that match the most cases (on par with organizational autonomy in FI). To conclude (similarly as in Stransky-Can, submitted), the relationship between NPM and NG is one of simultaneous occurrence in all three countries’ university policies. The understanding of these paradigms mirrors for AT and FI in financial autonomy with a tendency towards NPM and in staffing/academic autonomy, respectively, with a tendency towards NG. Both findings support the hypotheses that supportive paradigms in HE policy (for example, NG) are not necessarily less valued than competitive paradigms (for example, NPM), which alters the possibilities for EG to appear in HE steering (governance).

### On Theorizing on Differences Between External and Internal Perceptions of Governance

First, we look at means of information transmission from macro- to meso-levels and drivers for the interplay of the university management committees (and/or the relationship between horizontal and vertical autonomy) for AT, FI and SCO. In the concluding paragraph, the findings will show in a combination as to formulate hypotheses.

#### 1. Information Transmission from Macro- to Meso-levels

##### AT

University policies tend to favour the paradigm of NPM and the themes of financial and academic autonomy. In terms of theme drivers of a paradigm, financial autonomy mostly frames by NPM. An element of NPM is performance contract signed by strong^33^ rectorates: Performance agreements concluded between the Federal Minister for Education, Science and Research and each university, fix funding (excl. second- and third-party-funds) for a three-year-period. However, as universities in AT have limited possibilities in charging tuition fees, the financial autonomy ranking according to the EUA is, compared to the other autonomy themes and to FI and SCO, relatively low (EUA, 2021a, 44). So to describe the external perspectives range on the information of monies flows with some limitations for horizontal autonomy. Within the universities, i.e. from an internal perspective and focussing on vertical autonomy, universities have managed to professionalize their executive management (rectorates) as to strengthen its role for funding distribution^34^ within the institution (Assistant professor for research in education and further education, 2018). Even though financial autonomy reports as low, the strengthening of rectorates is obvious, and from a perspective of the NPM paradigm, this points to current momentum for top-down steering approaches in vertical autonomy.

##### FI

NG is the paradigm that frames most of the relevant cases in the Universities Act 2009. Academic autonomy is the theme that is the driver for NG. This includes substantial self-steering and self-organizing capacities, which is mirrored in the EUA autonomy ranking scores for academic autonomy being high as organizational and staffing autonomy and higher than in AT and SCO (UK from the EUA source). However, the Ministry of Education and Culture largely regulates requirements on degrees. In terms of limitations in vertical autonomy, an adjunct professor in higher education administration (2019) describes, “[T]he decree […] kind of determines which institutions can grant which degrees. […] Bachelors in law or bachelors in business studies or something like that, that said: These educational responsibilities are regulated in a decree level regulation.” In this example, the information is contained within a regulative act that puts ahead degree types (even though not numbers). Whereas academic autonomy reports as high, the possibilities for bottom-up steering approaches in vertical autonomy concerning content seem to be rather limited.

##### SCO

Here, too, university policy slightly favours the paradigm of NG. Organizational autonomy is clearly the dominant theme without showing a tendency towards a paradigm. Meanwhile, this theme also shows as the one for most room for manoeuvre in horizontal autonomy, compared to other autonomy themes and to AT and FI (EUA, 2021a, 41; 44; 47; 50). In contrast to the above presented cases, universities in SCO go as far as from vertical autonomy designs, the horizontal autonomy arrangements develop (representative SCO). Note that universities in SCO were the first movers among the compared countries when it comes to mergers.^35^ In this sense, information transmission from the meso-levels to the macro-level is concerned, which is a further indication for NG as there are networks explicitly focussing on forms of organizational learning. Therefore, the explicit formulation of a “regulatory agenda” (representative SCO) is of interest for both actors in governance and steering, also concerning other autonomy themes such as academic autonomy: “[U]niversity will tell you and government – that’s what they are concerned about is student access”. (ibid.) However, top-down articulation is present at a distance from government, here within the Scottish Founding Council: “There’s a lots of pressure, there’s funding agreements, funding pressure on that. [… W]hat it means is articulation. And they [universities] have a target, set by the [Scottish Founding] Council.” (ibid.)

#### 2. Interplay of the University Management Committees

##### AT

Academic autonomy is the theme, which matches the most cases, behind financial autonomy. In the EUA ranking, it scores somewhat higher than financial autonomy (but behind organizational autonomy – EUA, 2021a, b, 41; 50). Within an understanding of academic autonomy transforming into an understanding of academic freedom, the polity framework broken down into a politics meaning suggests: “[D]iese Perspektive des Freiheitsgrades für Forschung und Lehre, […] das ist etwas, wo offensichtlich alleine nur eine Institutionsautonomie nicht ausreicht, weil ich hier im Prinzip auf drei Ebenen diese Wechselwirkung habe, nämlich auf der Ebene der Individuen, des Systems und der einzelnen Institutionen” (assistant professor for research in education and further education, 2018). As to cover the micro-levels, it becomes clear that following the top-down policy logic of performance agreements (for governance) and target agreements (for steering) for the interaction of macro- and meso-levels, academia needs to integrate to a certain extent into an organizational framework. Faculties must be the central layer that takes the strategic decision of supporting an individual research grant application, under the perspective of chances for success. Such a decision at the meso-macro-level, i.e., the rectorate, would be inappropriate (Assistant professor for research in education and further education, 2018). It stays with the rectorate, however, nominated by the senate and elected by the university council, for being responsible for signing up a possible granting contract. The rectorate, in this case, reports to the university council and to the Federal Ministry of Education, Science and Research. The rectorate is today in a position to formulate on institutional differentiation^36^ (Member of the Federal Ministry of Education, Science and Research, 2018).

##### FI

The second-strongest theme in the Finnish Act is financial autonomy. Much of the debates on this theme have emerged from institutional differentiation in teaching within the universities of applied sciences sector, forced by budget cuts (Assistant professor for research in education and further education, 2018). Note that for universities too, as indicated for the universities of applied sciences, financial autonomy ranks relatively low (EUA, 2021a, 44). Under consideration of formula result from public funding and its addition from the performance agreements, the board of the university decides on the budget. This committee makes up, among others, of staff representatives, who might pull the break for the rectorate in leading the operations of the university. The approach from horizontal autonomy now seems to be: “So it’s [scientific publications] quite a strong instrument in steering. […] Even though that it has been denied by the ministry, you can see this trickling down to individuals. [… T]here are other ways for ensuring that, in a way, you know, there is less individual freedom that there would have been, say, 20 years ago or 30 years ago” (Adjunct professor in higher education administration, 2019). Funding might push towards academic core functions at the institutions, in this case on research. A cross-connection to effective steering at meso-levels is, again, not easy to find. Although universities received more autonomy in 2009, “the economical autonomy is not very strong due to direct guidance from the ministry” (Representative of the University of Helsinki, 2019). Funding formula application comes with auditing features: “So it requires a legal change to save the accreditation round. The accreditation is just a requirement that they have to be evaluated or audited by a regular basis, but there is no, for example, program accreditation” (Adjunct professor in higher education administration, 2019).

##### SCO

Academic autonomy matches with some cases for these universities. Compared to other themes, it reports relatively low autonomy scores for the UK (EUA, 2021a, 50). The interplay of academic and funding autonomy illustrates as well as the momentum for academic change coming from funding: “[T]here are lots of schemes and there where funded hubs to bring people to look into universities. And that if this is a level to coming in, you have to adapt your teaching. And then the system has to change, the university has to change their courses they are offering for people that where successful” (representative SCO). As the university governing body has to approve funding, it therefore brings the academic board under pressure, which is under discharge of the body. This comes with financial autonomy that also reports as relatively low within the autonomy scores (EUA, 2021a, 44). Here, the body might build on European initiatives like the Bologna process (member of the Federal Ministry), which also comes into the academic board through the university staff members (assistant professor for research in education and further education, 2018). “[T]he way we work is not covered by a legal framework. [… S]o, there might be a strategic driver for something or institutional practice and people agreeing to work with this thing” (representative SCO). For sure, university steering in SCO builds on government influence, too, despite the overall high autonomy scores in international comparison: “Within the Scottish system, even if you have some contribution from student, the whole quality direction is still about government and social policy leading at the university sector.” (ibid.)

#### 3. Hypotheses

From 1. **hypothesis z** formulates: There are different operating modes of communication at the macro- and at the meso-level, serving for different informational needs for governance and steering purposes. The intensity of these differences are more accentuated for AT and FI and less accentuated for SCO.

On the grounds of 2, we formulate **hypothesis y**: Within the institutions (as well as in the state), the old steering modes are prevailing for a certain time, to a certain extent. Impulses from the outside (institutional differentiation as a system-overarching vehicle [Wolff at “The Universities between Humboldt and Bologna”, see footnote 9], “Bologna”, both as concentrated macro-inputs – member of the Federal Ministry), might, in the short run, on these grounds be criticized (auditing from stimulated debates on efficiency – ibid.; SCO and FI both with mergers – for FI [Scandinavia], assistant professor for research in education and further education (2018); he would classify mergers as an early-move towards NPM; “mergers for stronger insitutions”: adjunct professor in higher education administration, 2019) or even blocked (strong reproductive capability, because of research). Blockades might be resolved within institutional autonomy, for example, with knowledge-democratic arrangements (see above, the “Research Questions” section).

### On the Sufficiency of NPM, NG, and on the Alternative Paradigm of Epistemic Governance

Table 1 shows some space within the legal framework to identify paradigms that go beyond NPM and NG. As proposed in the “Understanding the Frames of University Autonomy” section, there could be a critical institutional combination of NPM- and NG-approaches as to rely on an explicit understanding of the dynamics that form academic work, namely EG.

The challenge to overcome might be that there shall be not only “strong” rectorates, as outlined by the reforms in 2004 for Austria and 2009 for Finland, but also supported ones. This can mean organizational restructuring, as FI (and, earlier, SCO) has shown: “And it was that strive for stronger institutions that caused kind of like policy intention that we need stronger institutions by merging them to get bigger units and to give these units more autonomy. To kind of find their own way and find sort of giving them more kind of stronger autonomy they might better sort of national needs” (adjunct professor in higher education administration, 2019). Continued trust into welfare state (re-)configuration paralleled by trust into the quality of particular solutions for the university sector accompany such policy outputs, what might distinguish FI from AT. A politics-sensitive handling of full autonomy as in FI, in a sense of also negotiating big deals such as mergers, could FI make a leader in university policies (Assistant professor for research in education and further education, 2018) in the last decade. This might hold also true for organizing university administration in a sense of “governance light” under the thrive for orienting science towards international markets (ibid.).

A comparable approach towards politics, with concretizing potential policy dealers in the academic sphere, emphasizes for SCO, including strong student engagement: “I mean, the thing with the Scottish sector, the other thing that drives the way we work […]: the collegial way we work within it. So, we work towards the sector. [… V]ice rectors of learning and teaching […] learning from each that is very collective. But they also recognize that power of speaking with one voice. […] And they [the government] take that collective voice. […] They [universities] see to collectively respond to government agenda. [… I]t is a very collaborative sector” (representative SCO). As university policies in SCO are relatively strongly oriented towards NG (see above in “Results”), space for alternative paradigms within the HE institutions remains and is to use, especially within large traditional ones: “[T]hat makes negotiations different—depending on how much you have from government and how much you have from other sources. […] So when they [universities] are looking at how much [income] they need – they need some government and so much depends on their international profile.” (ibid.) Especially for traditional institutions, it becomes clear that these profiles are a matter of interacting macro- and micro-levels, therefore, involving the epistemic base.

In AT, switching from UOG 1993 structures (to break with “etatistischer Tradition”, member of the Federal Ministry; first universities of applied sciences as an institutional alternative have been founded) to UG 2002 rectorates (and “their” [?] deans) has been a very swift move with a clear autonomy market nexus. In contrast to FI with a gradual approach in autonomy discussion and consensus orientation, AT has oriented university policy towards administrative reform, with higher education evolving towards a citizen right (member of the Federal Ministry) functioning as an international umbrella term. In this sense, university policy has been ahead of institutional steering and strategy formulation, also in international comparison: “[O]hne UG wäre das österreichische Universitätssystem das altmodischste in ganz Europa, grosso modo”. (ibid.) In academic autonomy, reforms in 1997 have already altered law-bound curricula formulation towards approaches of peer review for teaching staff, learning devices and curricula. Note that peer review can be used as a tool for EG, namely when it is recognized as a quality enhancement (not just as a control) instrument. Of course, many university policies have adjusted meanwhile as to AT losing its frontrunner-position (ibid.), as the EUA autonomy scorecard underlines.

The deflation of configuring the NPM orientation in university policy reforms, internationally reflected as a systemic disadvantage for AT (Assistant professor for research in education and further education, 2018), has left space for adjustments at the meso-levels. For example, deans have been strengthened (Member of rectorate of an Austrian university, 2019), acknowledging the problems of early full autonomy (engaging with agency from the lower meso-/the micro-levels, above all the core faculty), with solutions for standstill or non-implementation situations demanding management engagement (integration of new administrative units, see also hypotheses y). This includes questions of the selection of meso-level management staff. It also includes how it is acting and what is happening with this staff after resigning from its function (Assistant professor for research in education and further education, 2018). However, answers to these questions have to come from agency-taking institutions and their actors. As a precondition, state targets have to formulate. The Austrian University Development Plan existing since 2016 may serve as an example. This is to organizationally support institutions: “Der Anspruch einer modernen Universität ist ja, dass sie sich überlegt, was sie tut. Das hat eine Universität alter Prägung ja nie getan, als Universität. […] Sondern in Wahrheit waren das an den Universitäten Sammelsurien von Einzelpersonen oder bestenfalls Arbeitsgruppen” (member of the Federal Ministry).

#### Hypotheses

##### Hypothesis x

 In the early phase of full autonomy, universities in AT have not been able to count on institutional strategic cooperation with other universities. This was due to lacking EG, i.e. (top-)administration has not been aware of academic cooperation formats within their institution. Meanwhile, awareness is developing, also because of increasing support for institutions.

##### Hypothesis w

 AT university policy implementation is control-oriented—“[t]atsächlich ist [die Idee von der Leistungsvereinbarung und von der Autonomie] auf der Verhaltenskontrolle stehen geblieben.” (member of the University for Continuing Education Krems; similarly, assistant professor for research in education and further education, 2018), whereas FI is trust-oriented. EG, therefore, proves as a multi-faceted alternative/additional paradigm for steering, i.e. executing vertical institutional autonomy. As Table 1 illustrates for AT: Why has the UG become much more extensive, compared to the UOG 1993? Was it engagement from universities’ level? What was the reason for this engagement?

Meanwhile, there is potential for EG interpretations within the university policies of AT, FI and SCO. For the first case, university founding is concerned, as § 120 (7) of the Universities Act 2002 says that professors, staff and students shall compose the foundation committee. Note the analogy to student engagement in SCO. There, § 1 (2) of the Higher Education Governance (Scotland) Act 2016 ensures the lay member of the governing body to guarantee a balance of authority between the body and the principal. As another example where the theme of organizational autonomy is matched, eventually within EG, the Universities Act 2009 of FI says in § 65 (2) that university collegium is responsible for discharging the board members and the rector of a university.

## Discussion

As already described in the “Outline, Background” section, university steering cannot work without the core faculty driving the “three missions”: Institutions shall enable to a broad understanding of the “core”, including staff and, to a certain extent, students. NG has good preconditions, as knowledge democracy is an established concept (see the “Research Questions” section). When looking at university policies, NG is currently expanding in AT and SCO. For the first country, NG seems to be similarly new on such as NPM has been in the university organizational act of 1993. Meanwhile, public administration is adjusting to NPM—if seen as an evolution in paradigm application, university policy becomes a pioneering field for NG (with EG in reality maybe coming under the wheels of both established paradigms in AT). SCO shows signs of late NPM application as universities there act within their most traditional functions, NG strongly represents,^37^ not within a thematic context, though. This leaves open SCOs direction towards an understanding of a knowledge state. Nevertheless, it shows system competitiveness that might go as far as there is agreed-on internal differentiation of and international harmonization for the universities sector (Table 2).

**Table 2:** Table of comparison for AT, FI and SCO

	**AT**	**FI**	**SCO**
**Welfare state type**	Conservative	Social democratic	Liberal
**Redistribution capacity (theoretical)**	Moderate (depending on occupational fields)	Strong (universal labour market)	Weak (residual safety net)
**Current redistribution capacity (see note)**	Moderate	Strong	Weak
**Stakeholder included into legislative processes**	Under average, increasing	Average, decreasing	Above average, increasing
**NPM-narrative**	Outcome-oriented steering	Dialogue and change process	Issue-oriented coordination
**NPM**	Slightly dominant (decreasing)—> pendulum swings back to normal	Dominated (increasing!)—> with liberal elements	Dominated (!)—> this comes unexpected
**Trust into the education system**	71% agreements (declining)	84% agreements (increasing)	71% agreements (UK—increasing after slump)
**Interested into science in 2013**	Under average	Above average	Above average (UK)
**Institutional differentiation**	From the beginning of the 1990s (universities of applied sciences)	Has begun before period of investigation	Has begun before period of investigation, but “very stable” compared to England; colleges “engaging with universities”
**Mergers**	None	First, universities of applied sciences; then, universities	(very) early, begun with institutional differentiation, already 2003
**State funding for universities currently**	Increasing	Stagnating	Decreasing
**Access for students**	“Matura” (state exams); fees for studies taking longer; access regulation for certain fields	Results from state exams; fees for international students; access regulation through decree	Results from school leaving exams; fees, for undergraduate students from government (restricted)
**Prevalent funding sources**	Public (slowly declining)	Public (declining)	Public (considerable private in research)
**Entered the EU in**	1995	1995	1973
**Unique selling propositions**	Private universities	No funding for study places (“There is no baseline”.)	University names not included in university acts

Source: The author based on EUA (2021b); OECD (2019); Adjunct professor in higher education administration (2019); Stransky-Can (2019a); Representative SCO (2018); European Commission (2013); Esping-Andersen (1990)

Note: A high gross debt ratio indicates a low redistribution capacity (and vice versa)

NPM and NG are not all encompassing. As an alternative paradigm, EG is an established concept (see the “Theorizing on Differences Between External and Internal Perceptions of Governance” section). Matches between the paradigm and university policy are present, but there is need for more research for a concrete conclusion, as outlined, for example, with finding an accentuated understanding of all three paradigms from their contexts of identification. This approach names with “inner paradigmatic accentuation” (Stransky-Can, submitted). For connecting NPM and autonomy evolution, results for FI university policy show a slightly increasing popularity for NPM between 1997 and 2016. The observation of an increase also confirms for the EUA autonomy scores as compared for 2010 and 2016. In contrast, in AT university policy, NG is increasingly used, which comes with a coincidence of somehow reduced autonomy scores between 2010 and 2016. A (less accentuated) increase of NG also holds true for SCO university policy, with this country (as represented within the UK) stagnating on a high level within the autonomy scores.

When we compare the EUA (2021a) autonomy scorecard framework with the earlier publication of Aghion et al. (2007), a differentiation between core and peripheral autonomy dimensions may be appropriate for further analysis of horizontal and vertical autonomy in university politics. As later, the dimensions of academic and organizational autonomy create, whereas Aghion et al. (2007) already covered the financial and staffing dimensions. As for the core autonomy dimensions: Academic autonomy fits investigations of trade-offs between the institutional and the academic levels, whereas organizational autonomy adds ramifications to the state-meso- and meso-meso-relationships.

## Footnotes

1 Parts of this section have been included in a public presentation of the doctoral thesis proposal at the University of Vienna in 2017.

2 Governance means the interplay of the state and universities, “understood as designing the relationship between the state and the institution”, whereas steering concerns overall universities management (from top to low) “as structuring the relationships between different stakeholders groups within an institution, i. e., the organization as a political system” (Stransky-Can, 2019a, 3).

3 The UG in its English translation is available at https://www.ris.bka.gv.at/Englische-Rv/ (retrieved on 2021–07-29). Do not confuse the UOG (1993) with the UOG (1975).

4 (Inter-)Institutional mergers have been performed in this country since before passing current legislature, one of the most prominent examples being Aalto University in Helsinki (created at the beginning of 2010 from the School of Economics, the University of Technology and the University of Art and Design, all located in Helsinki). Consequently, the Research Institute of the Finnish Economy (2017) is proposing the general possibility for universities to charge “moderate tuition fees”. Indeed, the possibility to charge considerable fees from foreign students has extended financial autonomy for universities in FI.

5 As a possible outcome of these reforms, we see the positions of FI in the European University Association (EUA) autonomy rankings for 2010 and 2016: FI universities rank higher more recently.

6 Concretely for the cases of the UK and France. Similarly, on the UK, Paradeise et al. (2009) explain how HE institutions went ahead in autonomy. This still mirrors in the EUA (2021a, 41; 44; 47; 50) autonomy ranking where the UK is the only large country being in the forefront group for all measured dimensions.

7 Parts of this section have been included in a public presentation of the doctoral thesis proposal at the University of Vienna in 2017.

8 Later, Österreichischer Wissenschaftsrat (2015, 28) provides for a similar (theoretical) autonomy definition for HE institutions.

9 For FI: “First, establishing more institutions and then to create a new universities sector, the universities of applied sciences, and then kind of softer steering the sector through structural development. So, through these mergers and sort of changing, in a way, changing the instrumental steering from the streamline to a more performance-based management which means that more through the outputs” (adjunct professor in higher education administration). Taken as that, this statement points towards systemic change management (a term taken from Birgitta Wolff at the event “The Universities between Humboldt and Bologna” at the Danube University Krems, https://www.donau-uni.ac.at/en/news/news/2021/the-universities-between-humboldt-and-bologna.html [retrieved on 2021–07-29]).

10 For its most recently published form in English up to an amendment in 2016, see https://finlex.fi/en/laki/kaannokset/2009/en20090558_20160644.pdf (retrieved on 2021–07-29).

11 See https://www.legislation.gov.uk/asp/2016/15/contents/enacted?view=plain (retrieved on 2021–07-29).

12 Note that for the period under investigation, AT was the first to decide on a new universities act in 1993. In 1997, FI has followed, whereas early empirical analysis shows that the 1992-act of SCO does not reveal enough cases for comparison in question a. Therefore, the first relevant act for SCO is identified with the Further and Higher Education (Scotland) Act 2005.

13 Substantial parts of this section come from Stransky-Can and Campbell (submitted, 2–3).

14 Framing analysis is a method to match a pre-defined theme with data and, subsequently, to locate it within a framework of meaning making—the frame.

15 Note that the European University Association (EUA, 2021a) calls the four cited aspects as “key dimensions” of university autonomy. The reason for using the term of “dimensions” for horizontal and vertical (and scientific) autonomy here comes from the approach of first fixing the field of research that lies predominantly in horizontal institutional autonomy and, second, modelling the methodology for working in the field, whereas the framing analysis is searching for themes in university policy. Here, for example, academic autonomy would be one aspect of university autonomy under which we can understand university policy formulation, which directly concerns the dimension of horizontal autonomy.

16 “[T]uition fees are made to create markets” (representative SCO). Interestingly, SCO goes far compared to AT and FI (where a small fraction of students is affected) when its universities may charge tuition fees for all graduate studies. Nevertheless, the government of SCO reimburses undergraduate student fees.

17 This sub-section comes partly from Stransky-Can and Campbell (submitted, 4).

18 AT, rectorate for leading the operations; university council as a (partly externally appointed) supervisory board; senate for academic affairs (confirmation of tenure-track accomplishments; directions of teaching; participation in professorship appointment procedures); FI, rectorate for leading the operations; internally elected university board decides on academic affairs and as a supervisory board; SCO, principal for leading the operations and academic affairs; university governing body as a committee of transmission between academic and administrative affairs; academic board for academic affairs.

19 Stransky-Can (2019b) proposes the terms of horizontal and vertical institutional autonomy for university policy analysis. More theoretically, see also Campbell and Stransky-Can (2020).

20 See the “Operationalizing NPM, NG and Their Understandings” section for an occupation of these themes that fits management operationalization purposes.

21 Note that the state is applying the narratives of NPM and NG, and it is interesting to study to which extent and in which combination universities are implementing them (and whether alternative paradigms might develop from these implementation processes).

22 Global budgets mean that institutions are free to distribute state funding internally as they see fit.

23 Moreover, as relics instead of traditions, law documents are classified by Treib (2010, session 3, 3) as having a higher epistemic value than, for example, press releases.

24 The period of interviewing spans from April 2018 to October 2020, with a dozen interviews in total, see the reference list for a selection.

25 Parts of this sub-section come from Stransky-Can and Campbell (submitted, 1; 3).

26 For sector-overarching conclusions, also including other governance fields, see Caraynnis and Campbell (2021).

27 It is possible that some matches are the same for both acts in the respective countries. For the reasons explained in this paragraph (offsetting or additive regulation), however, their number must be low. It is, therefore, adequate to treat the newer law and the older law as separate entities.

28 Political science distinguishes between polity, which is the (constitutional) amalgam on which institutions are built upon; politics, which is the layer of interplay between institutions and their actors; and policy, which is the outcome of this interplay (in the form of law, for example).

29 Although visible differences between AT, FI and SCO remain in financial autonomy for universities (EUA 2021a, 44), a common feature are global budgets.

30 This means that markets are more traditional institutions than the nation states. Governance without state politics, including public (state and universities) administration, would be unthinkable in welfare states, however.

31 The description of the results presentation in this sub-section comes from Stransky-Can (submitted).

32 The non-popularity of NPM in SCO and its differences in governance approaches to England highlights from the perspective of a senior member of Scottish student representatives.

33 The way to hypothesis x will further discuss what this attribute can mean.

34 Note that financial autonomy for universities in AT goes as far as they are free how to distribute internally their monies from different funding sources (even though the sums in the performance agreements mostly show as a result from formulae).

35 https://researchportal.bath.ac.uk/en/studentTheses/exploring-critical-factors-in-the-university-of-the-west-of-scotl (retrieved on 2021–06-24).

36 What this means for universities in AT, in relation to internal integration, is formulated within hypothesis x.

37 This points to the question as how to isolate NG from traditional steering approaches.
